# The effect of intrauterine growth restriction on Ca^2+^‐activated force and contractile protein expression in the mesenteric artery of adult (6‐month‐old) male and female Wistar‐Kyoto rats

**DOI:** 10.14814/phy2.13954

**Published:** 2018-12-27

**Authors:** Michael J. Christie, Tania Romano, Robyn M. Murphy, Giuseppe S. Posterino

**Affiliations:** ^1^ Department of Physiology, Anatomy and Microbiology La Trobe University Melbourne Victoria Australia; ^2^ Department of Biochemistry and Genetics La Trobe Institute for Molecular Sciences La Trobe University Melbourne Victoria Australia

**Keywords:** Arteries, calcium activated force, chemically permeabilized, intrauterine growth restriction, vascular responsiveness

## Abstract

Intrauterine growth restriction (IUGR) is known to alter vascular smooth muscle reactivity, but it is currently unknown whether these changes are driven by downstream events that lead to force development, specifically, Ca^2+^‐regulated activation of the contractile apparatus or a shift in contractile protein content. This study investigated the effects of IUGR on Ca^2+^‐activated force production, contractile protein expression, and a potential phenotypic switch in the resistance mesenteric artery of both male and female Wistar‐Kyoto (WKY) rats following two different growth restriction models. Pregnant female WKY rats were randomly assigned to either a control (C; *N* = 9) or food restriction diet (FR; 40% of control; *N* = 11) at gestational day‐15 or underwent a bilateral uterine vessel ligation surgery restriction (SR;* N* = 10) or a sham surgery control model (SC;* N* = 12) on day‐18 of gestation. At 6‐months of age, vascular responsiveness of intact mesenteric arteries was studied, before chemically permeabilization using 50 *μ*mol/L *β*‐escin to investigate Ca^2+^‐activated force. Peak responsiveness to a K^+^‐induced depolarization was decreased (*P *≤* *0.05) due to a reduction in maximum Ca^2+^‐activated force (*P *≤* *0.05) in both male growth restricted experimental groups. Vascular responsiveness was unchanged between female experimental groups. Segments of mesenteric artery were analyzed using Western blotting revealed IUGR reduced the relative abundance of important receptor and contractile proteins in male growth restricted rats (*P *≤* *0.05), suggesting a potential phenotypic switch, whilst no changes were observed in females. Results from this study suggest that IUGR alters the mesenteric artery reactivity due to a decrease in maximum Ca^2+^‐activated force, and likely contributed to by a reduction in contractile protein and receptor/channel content in 6‐month‐old male rats, while female WKY rats appear to be protected.

## Introduction

Epidemiological and experimental studies have recognized that IUGR or the failure of an infant to achieve their genetic potential for growth are prone to developing diseases in later life, including cardiovascular diseases such as hypertension and coronary heart disease (Barker 1994; Gluckman and Hanson 2004; McMillen and Robinson 2005). IUGR is a manifestation of several maternal, paternal and fetal factors, arising from genetic or environmental issues, resulting in poor growth of the developing fetus (Peleg et al. 1998).

A common cause of IUGR in Western society is uteroplacental insufficiency, occurring when remodeling of placental spiral arteries is incomplete thus, reducing both nutrient and oxygen availability to the developing fetus (Khong et al. 1986; Henriksen and Clausen 2002). A bilateral uterine vessel ligation surgery restriction (SR) is often used to mimic this condition in animal studies (Wlodek et al. 2005). In the developing world, maternal malnutrition is the major cause of IUGR, resulting from lasting nutrient deficiency of the pregnant mother negatively affecting the growing fetus (Bergmann et al. 2008). Maternal dietary manipulations, such as a food restriction (FR) diet, are used to imitate this condition in animal studies (Williams et al. 2005a,b). Several studies utilizing these IUGR animal models have reported changes to vascular smooth muscle responsiveness (Williams et al. 2005a; Tare et al. 2012), endothelial dysfunction (Goodfellow et al. 1998; Leeson et al. 2001) and increased arterial wall stiffness (Khorram et al. 2007), which contribute to the development of cardiovascular disease in adulthood.

Evidence of an altered extracellular vascular responsiveness to certain vasoactive chemicals has been widely recognized in several IUGR rat models (Williams et al. 2005a; Anderson et al. 2006; Tare et al. 2012 to name a few). However, it is unclear whether these observed changes in vascular sensitivity are driven by changes in receptor number/activity or by changes in downstream events that lead to force development; specifically, the Ca^2+^‐regulated activation of the contractile apparatus. Vascular smooth muscle contraction is primarily controlled by the level of intracellular [Ca^2+^] which regulates the balance of myosin light chain kinase (MLCK) and myosin light chain phosphatase (MLCP) activities, biochemically controlling the Ca^2+^–tension relationship through increasing or decreasing phosphorylation of the myosin light chain (MLC) (Kitazawa and Somlyo 1990; Kitazawa et al. 1991; Somlyo and Somlyo 2003). Reducing MLCP activity while increasing MLCK activity through a rise in [Ca^2+^]_*i*_ tilts the balance toward generating extra force.

Vascular smooth muscle cells (VSMC) are not terminally differentiated (Owens 1995), they retain enormous plasticity to fulfil several functions, switching between a contractile and proliferating/synthetic phenotype (Owens et al. 2004; Salmon et al. 2012). A contractile phenotype is characterized by an upregulation of smooth muscle markers (Owens 1995; Hungerford and Little 1999) which include contractile and cytoskeletal proteins (e.g., *α*‐actin and smooth muscle myosin heavy chain (Myh11)), allowing for the regulation of vascular tone. However, the proliferating/synthetic phenotype downregulates many of those contractile proteins, and synthesis’ more structural and extracellular matrix proteins including tropomyosin 4 (TPM4), *β*‐actin, collagen type I and III, which helps repair injured vessels and allows for vascular adaptation in diseased states (Jain 2003; Yoshida et al. 2008). The exact mechanisms for activating phenotypic switching is complex and highly variable in different diseased states and often involves Ca^2+^ signaling pathways (House et al. 2008). In models of IUGR, little is known about the expression of these key protein markers associated with phenotypic switching.

Therefore, the aim of this study is to examine the effects of IUGR on Ca^2+^‐activated force responses in chemically permeabilized artery segments and to further investigate a potential shift in abundance of important protein markers associated with phenotypic switching in adult (6‐month‐old) male and female WKY rat mesenteric arteries. Furthermore, because a systematic examination of the effects of different models of IUGR are not typically made, in this study, we used two different growth restriction models (FR and SR) to compare the effects of a diverse approach to inducing IUGR on vascular smooth muscle responsiveness.

## Materials and Methods

### Ethical approval

All experimental procedures were approved by the La Trobe University Animal Ethics Committee (AEC no. 14‐22) and conform to the National Health and Medical Research Council of Australia guidelines.

### Animal models

WKY rat dams were housed at 22°C on a 12:12 h light/dark cycle with ad libitum access to standard rat chow and water. Dams were mated overnight after they were identified to be in proestrus (Wlodek et al. 2003, 2005; O'Dowd et al. 2008). Pregnancy was confirmed by sperm present in the vaginal smear obtained the following morning; this was considered day 1 of gestation. Pregnant dams were then randomly designated to either a FR diet or uteroplacental insufficiency protocol to induce IUGR.

Rats designated to the FR protocol were housed individually with food intake measured daily throughout pregnancy. On gestational day 15, 11 dams were randomly selected to undergo a 60% FR diet (based on each dam's average daily food intake prior to day 15; *N* = 11); as previously described by (Williams et al. 2005a,b; Harvey et al. 2015). The nine remaining dams (untreated C; *N* = 9) had continuous ad libitum access to food. Following birth (term = day 22), all dams were given ad libitum access to food.

Pregnant rats designated to the uteroplacental insufficiency model were housed together until gestation day 18 before being randomly divided into a SC (sham surgery; *N* = 12) or SR (uteroplacental insufficiency; *N* = 10) group, before undergoing a bilateral uterine vessel ligation surgery, as previously described (Wlodek et al. 2005, 2007; O'Dowd et al. 2008). Litter size was not equalized between groups, as it has been demonstrated that reducing litter size can impair maternal lactation and postnatal growth, and thus do not represent adequate controls (O'Dowd et al. 2008; Wadley et al. 2008; Wlodek et al. 2008).

### Mesenteric artery isolation and assessment of functionality

Offspring were weighed on postnatal day 1 and weaned at 5‐weeks of age with sexes separately housed. From each litter, a single male and female offspring was used for both physiological and biochemical experiments at 6‐months of age; which represents an adult human (~35–40 years old). On the experimental day, offspring were killed by overdose of isoflurane (4% v/v) inhalation in a glass chamber, with the heart being rapidly excised to ensure death. A large portion of the intestine, cut at the proximal end near the pylorus and distal end close to the ileo–coecal junction, was excised and pinned out onto a Sylgard coated petri dish placed in cold standard physiological saline solution (PSS; containing in mM: 10 HEPES, 150 NaCl, 3 KCl, 2.5 CaCl_2_, 1 MgCl_2_ and 5.5 glucose; pH 7.3), with constant 100% O_2_ aeration whilst the mesenteric artery was isolated. All chemicals were sourced from Sigma‐Aldrich (St Louis, USA) unless otherwise specified.

A small ~2 mm long section of 3rd order mesenteric artery was separated from the vein and placed in the single wire myograph system (320A, Danish Myo Technology A/S, Denmark) containing warmed (37°C) PSS. Two 40 *μ*m diameter wires were threaded through the lumen of each artery, with length of each preparation being carefully measured (Mulvany and Halpern 1977). Each artery underwent an equilibration period (30 min) before being normalized, as previously described (Mulvany and Halpern 1977; Anderson et al. 2006). The arterial preparation was equilibrated for an additional 30 min, followed by a standard start procedure, as previously described (McIntyre et al. 1998; Anderson et al. 2006), before the functionality of each mesenteric artery was investigated; note, endothelium integrity was checked through responsiveness to 10^−6^ mol/L acetylcholine. Briefly, the intact preparation was exposed to K^+^‐PSS (equimolar substitution of NaCl with KCl in PSS) which initiated a K^+^‐induced depolarization contractile response. The vessel was also exposed to increasing concentrations of PE (10^−8^–10^−4^ mol/L) to determine the *α*_1A_‐adrenergic receptor‐mediated contractile responsiveness. Maximum responsiveness to K^+^‐PSS and PE was measured by normalizing peak force (mN) to the arteries length (mm).

### Determining Ca^2+^‐sensitivity and maximum Ca^2+^‐activated force response

To ascertain the properties of the contractile apparatus specifically, the same artery preparation was then chemically permeabilized with 50 *μ*mol/L *β*‐escin in a heavily Ca^2+^‐buffered K^+^‐EGTA solution (solution A; containing in mmol/L: 50 EGTA, 90 HEPES, 10.3 Mg^2+^_total_, (1 free Mg^2+^), 8 ATP _total_, 10 creatine phosphate, 125 K^+^, 36 Na^+^; pH 7.10 ± 0.01) for 30 min at room temperature (22°C), as previously described by Satoh et al. (1994). Note: we have conducted numerous control experiments to ascertain the optimal permeabilization conditions for this preparation (Christie 2018). Each artery was washed with solution A three times before a Ca^2+^‐response curve was performed, by exposure to a sequence of highly buffered Ca^2+^‐EGTA solutions with increasing levels of free [Ca^2+^] (pCa (−log [Ca^2+^]) 7.5–4.5), by mixing suitable volumes of solution A with a Ca^2+^‐EGTA solution (solution B: pCa 4.5) which was identical in composition to solution A with the exception that it contained in mmol/L: 8.12 Mg^2+^_total_ (1 free Mg^2+^), 49.7 Ca^2+^_total_ (see Stephenson and Williams (1981) for apparent affinity constants). As the level of free [Ca^2+^] increased there was a step‐wise increase in force which was used to create a force–pCa relationship for each chemically permeabilized arterial preparation. All intracellular physiological solutions contained 50 units per ml of creatine phosphokinase and 10 *μ*mol/L guanosine‐5′‐triphosphate as this has been previously shown to help maintain consistent force production throughout experiments (Akata and Boyle 1997).

The Ca^2+^‐sensitivity of the contractile apparatus was ascertained by normalizing all submaximal force responses elicited to low pCa solution, to the maximum Ca^2+^‐activated force, which was plotted against the pCa. A nonlinear regression, specifically single exponential association derived from equation 1 was used to fit the points of the curve for each preparation, using GraphPad Prism 6.01 (as described previously by Williams et al. (2017)).

(1) $$\begin{array}{cc} Y & =\text{Bottom}+(\text{Top}-\text{Bottom})/\\ & \;\left(1+10\left(\left(\text{LogEC50‐X}\right)*\text{Hill slope}\right)\right), \end{array}$$

where *Y *= *Y*‐axis; Bottom = smallest *Y* value at the bottom of the plateau (0%); Top = largest *Y* value at the top of the plateau (100%); EC_50_ = *X* value which represents half‐maximal (*Y*) response; *X *= *X*‐axis; Hill slope = gradient of the curve.

The pCa that elicits 50% of the maximum Ca^2+^‐activated force response (pCa_50_) and the Hill slope coefficient was collected from each fitted curve and averaged. These values were then used to create a representative curve. This approach was employed as it is better than plotting the overall mean data for each preparation and then fitting a single curve to the mean data, as the latter has the possibility of artificially skewing the fitted curve providing an incorrect assessment of the individual preparations responsiveness to Ca^2+^ (Lamb and Stephenson 1990, 1994; Posterino et al. 2000). The maximum Ca^2+^‐activated force was measured by normalizing the peak force (mN) to the arteries length (mm).

### Western blotting

A second ~4 mm long section of 3rd order mesenteric artery was taken from an adjacent arterial arcade to the previous preparation. The preparation was physically homogenized with a glass Dounce tissue grinder (2 mL; Sigma‐Aldrich) whilst frozen with liquid nitrogen, then chemically homogenized with 200 *μ*L of 1 × solubilizing buffer, containing: 0.125 mol/L Tris‐HCl, 10% glycerol, 4% SDS, 4 mol/L urea, 10% mercaptoethanol and ~0.001% bromophenol blue (pH 6.8) diluted (2:1 v/v) in a modified PSS solution (2.5 mmol/L CaCl_2_ removed and 2 mmol/L EGTA added). Preparations were stored at −80°C until analysed by western blotting using a protocol described previously (MacInnis et al. 2017).

Briefly, homogenized artery samples were loaded onto a 4–15% criterion TGX stain‐free protein gel (Bio‐Rad, Hercules, CA, USA). A 4‐point calibration curve of known volumes (2, 4, 8 and 16 *μ*L) was generated by loading a calibration mix, as previously described (Edwards et al. 2010; Murphy and Lamb 2013). Ultraviolet activation of the gel allowed visualization of total protein loaded and was subsequently analyzed using ImageLab 5.2.1 (Bio‐Rad). Separated proteins were transferred to a stable nitrocellulose membrane. Membranes were cut horizontally, and individual sections were probed with specific primary antibodies diluted in 1% BSA in PBS with 0.025% Tween incubated at room temperature for 2 h and 4°C overnight on rockers. The proteins of interest include: *α*_1C_ L‐type voltage gated Ca^2+^‐channel (Ca_V_1.2; rabbit polyclonal IgG; 1:1000; AB5156; lot no. 2710733; Merck Millipore, Germany), inositol trisphosphate receptor 1 (IP_3_R1; mouse monoclonal IgG; 1:100; L24/18; lot no. 437‐1VA‐71; NeuroMab, USA), MLCK (mouse monoclonal IgG; 1:1000; M7905; Sigma‐Aldrich, USA), myosin phosphatase target subunit 1 (Mypt1; rabbit polyclonal IgG; 1:1000 sc‐25618; lot no. C2415; Santa Cruz, USA), Myh11 (mouse monoclonal IgG; 1:10,000; sc‐6956; lot no. C1815; Santa Cruz, USA), *α*‐actin (rabbit polyclonal IgG; 1:10,000; ab5694; lot no. GR248336‐21; Abcam, UK) and TPM4 (rabbit polyclonal IgG; 1:1000; AB5449; lot no. 2742738; Merck Millipore, Germany); note: all antibodies were independently verified in our laboratory using tissue panels (Christie 2018).

Membranes were washed and the applicable secondary antibody applied, either goat anti‐rabbit IgG HRP (Pierce 31460; 1:20,000; ThermoFisher Scientific) or goat anti‐mouse IgG HRP secondary antibody (Pierce 31430; 1:20,000; ThermoFisher Scientific) for 1 h at room temperature. Membrane sections were then washed before being exposed to either highly sensitive enhanced chemiluminescent (Pierce) substrate or Supersignal West Femto (Pierce), imaged with ChemiDoc MP (Bio‐Rad) and analyzed, using ImageLab 5.2.1 (Bio‐Rad).

The 4‐point calibration curve was constructed, and the slopes of linear regression used to compare relative amounts of protein between unknown samples, as demonstrated previously (Mollica et al. 2009; Murphy and Lamb 2013). Using the calibration curve, the relative amount of total protein in each lane, and subsequent density of each band could be accurately measured by expressing each relative to the calibration curve. The relative amount of each specific protein could then be normalized to the relative total protein. These values were further normalized through dividing by the average of “control” sample densities, which is why “control” values will always equal 1 ± SD, whereas, “restricted” samples will increase/decrease relative to that value.

### Statistical analysis

Datasets are expressed as mean ± SD, with number of individuals denoted as *N*. Unpaired student's *t*‐tests were used to determine statistical significance (*P *≤* *0.05), unless otherwise specified. All statistical analyses and data fitting was performed using GraphPad Prism 6.01. Note that one‐tailed *t*‐tests were used to analyse data throughout this study, as previous studies have demonstrated a specific shift in contractile force in one direction (Williams et al. 2005a; Anderson et al. 2006; Tare et al. 2012).

## Results

### Offspring body weight

There was no difference in litter sizes between the untreated C and FR group (~10 pups each litter). Uteroplacental insufficiency resulted in a litter size which were approximately 50% smaller in comparison to the SC group (from 7 to 4 pups) at birth. The body weight of pups from each experimental group (males and females combined) on postnatal day 1 was significantly different from all other groups (*P *≤* *0.05; one‐way ANOVA with Tukey correction); SR pups were smallest (3.7 ± 0.6 g), followed by FR pups (4.1 ± 0.3 g), then SC pups (4.4 ± 0.4 g) and lastly untreated C pups were heaviest (4.7 ± 0.3 g). It is interesting to note, as others have found (Nusken et al. 2008), that even the sham operation (SC group) or the necessary direct exposure to anaesthesia, affects fetal development and resulted in significantly smaller pup sizes in comparison to the untreated C group.

By 6 months of age, all experimental male groups had similar body weights (C: 374 ± 14 g; FR: 369 ± 19 g; SC: 381 ± 16 g; SR: 374 ± 35 g), whereas, SR females remained significantly smaller than C (*P *≤* *0.05; one‐way ANOVA with Tukey correction), but were not statistically different to both FR and SC females (C: 219 ± 5 g; FR: 217 ± 9 g; SC: 214 ± 9 g; SR: 203 ± 20 g).

### Intact mesenteric artery reactivity and functionality responses

Resistance mesenteric arteries underwent a K^+^‐induced depolarization which initiated a contractile response to determine depolarization‐induced peak force (see Table 1). Both FR and SR male experimental groups had significantly reduced contractile responses compared to their respected control groups (C and SC). Within female experimental groups, peak responsiveness was not different (C vs. FR; SC vs. SR).

**Table 1:** Mesenteric artery responsiveness to a K^+^‐induced depolarization and PE‐stimulation

	C	FR	SC	SR
Male	(9)	(8)	(8)	(8)
Peak K^+^‐response (mN/mm)	8.73 ± 1.38	6.95 ± 0.49**	8.22 ± 1.50	6.14 ± 1.32**
Max PE‐response (mN/mm)	10.25 ± 1.39	9.94 ± 1.24	9.81 ± 1.46	8.01 ± 0.59**
pPE_50_ (−log M)	5.62 ± 0.20	5.69 ± 0.23	5.80 ± 0.34	5.60 ± 0.16
Hill slope	3.41 ± 0.72	3.56 ± 0.86	3.76 ± 1.31	3.63 ± 1.68
Female	(9)	(11)	(12)	(10)
Peak K^+^‐response (mN/mm)	6.94 ± 0.69	6.67 ± 1.33	6.25 ± 1.34	5.79 ± 1.66
Max PE‐response (mN/mm)	8.41 ± 1.23	8.85 ± 1.74	6.99 ± 1.70	6.89 ± 2.38
pPE_50_ (−log M)	5.61 ± 0.20	5.68 ± 0.22	5.64 ± 0.16	5.63 ± 0.08
Hill Slope	3.24 ± 0.92	3.68 ± 0.98	3.49 ± 1.69	3.02 ± 0.64

Data expressed as mean ± SD with number of individuals (*N*) shown in brackets. Significant difference (**P < 0.01; one‐tailed unpaired *t*‐test) between relevant control and restricted (C vs. FR; SC vs. SR) experimental groups.

Contractile responses to the *α*_1A_‐adrenergic receptor‐mediated agonist (PE) was measured in each experimental group (Table 1). Peak response to PE was significantly reduced in SR males compared to the SC experimental group, whereas no differences in peak force were discovered between FR and C males. Furthermore, peak PE‐response was unchanged between female experimental groups. PE‐sensitivity was unaffected by growth restriction in utero in both male and female experimental groups as determined by the pPE_50_ and Hill slope values shown in Table 1.

### Ca^2+^‐activated force responses in *β*‐escin permeabilized mesenteric arteries

**Figure 1:**
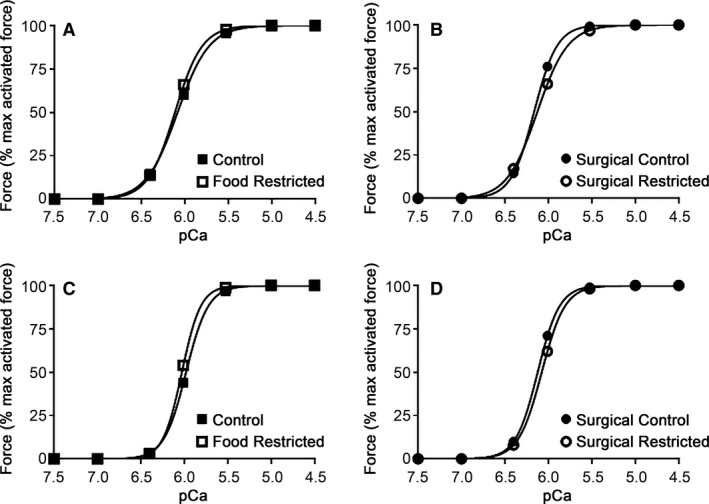
Representative force–pCa relationship. Individually fitted representative curve which closely follows the mean pCa_50_ and Hill Slope shown in Table 4 from (A) male control versus FR and (B) male SC versus SR. (C) Female control versus FR and (D) female SC versus SR. No statistical differences were present between experimental groups.

The Ca^2+^‐sensitivity and maximum Ca^2+^‐activated force was measured in each *β*‐escin permeabilized preparation by directly activating the contractile apparatus with an increasing concentration of free [Ca^2+^] (pCa 7.5 to pCa 4.5) heavily buffered as displayed in Figure 1. A representative force–pCa relationship which closely follows the average pCa_50_ and Hill slope value are shown in Figure 1. Table 2 shows the mean data. There were no apparent differences in Ca^2+^‐sensitivity between growth restriction models for both sexes as indicated by pCa_50_ and Hill slope. However, maximum Ca^2+^‐activated force (normalized to length of preparation) was significantly decreased in both growth restricted male groups (FR and SR) compared to their respective controls (C and SC). This reduced maximum Ca^2+^‐activated force was not observed in either female experimental groups.

**Table 2:** Ca^2+^‐sensitivity of the contractile apparatus and maximum Ca^2+^‐activated force in the mesenteric artery

	C	FR	SC	SR
Male	(9)	(8)	(8)	(8)
Max Ca^2+^‐response (mN/mm)	3.09 ± 0.41	2.72 ± 0.39*	2.91 ± 0.65	2.49 ± 0.24*
pCa_50_ (−log M)	6.08 ± 0.10	6.11 ± 0.17	6.16 ± 0.12	6.12 ± 0.15
Hill slope	2.39 ± 0.55	2.75 ± 1.34	3.23 ± 1.74	2.45 ± 0.73
Female	(8)	(11)	(12)	(10)
Max Ca^2+^‐response (mN/mm)	2.76 ± 0.31	2.83 ± 0.58	2.49 ± 0.53	2.77 ± 0.75
pCa_50_ (−log M)	5.97 ± 0.10	6.02 ± 0.10	6.12 ± 0.08	6.07 ± 0.05
Hill slope	3.25 ± 1.33	3.73 ± 1.56	3.30 ± 0.52	3.12 ± 1.26

Data expressed as mean ± SD with number of individuals (*N*) shown in brackets. Significant difference (*P ≤ 0.05; one‐tailed unpaired *t*‐test) between relevant control and restricted (C vs. FR; SC vs. SR) experimental groups.

### Mesenteric artery contractile protein expression

**Figure 2:**
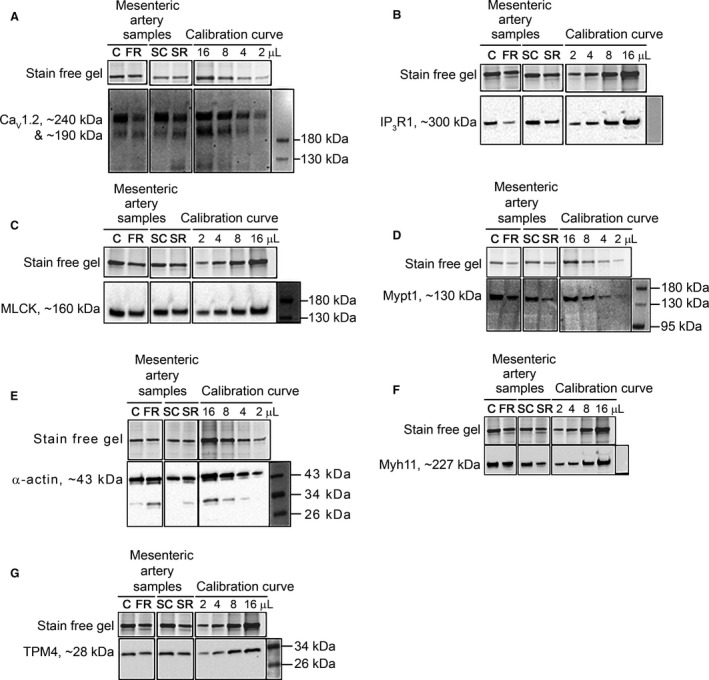
Representative western blots of the relative abundance of important contractile proteins from mesenteric artery samples of 6‐month‐old male offspring. Relative content (bottom panel) of (A) Ca_V_1.2, (B) IP_3_R1, (C) MLCK, (D) Mypt1, (E) *α*‐actin, (F) Myh11 and (G) TPM4 on western blot with the total protein indicated by the stain free gel (top panel) from control diet (C), food restricted (FR), surgical control (SC) and surgical restricted (SR) male offspring. Calibration curve with amount loaded (in *μ*L) specified at top in all panels. All lanes shown are from same gel. Mean ± SD values and statistical analysis displayed in Table 3.

**Figure 3:**
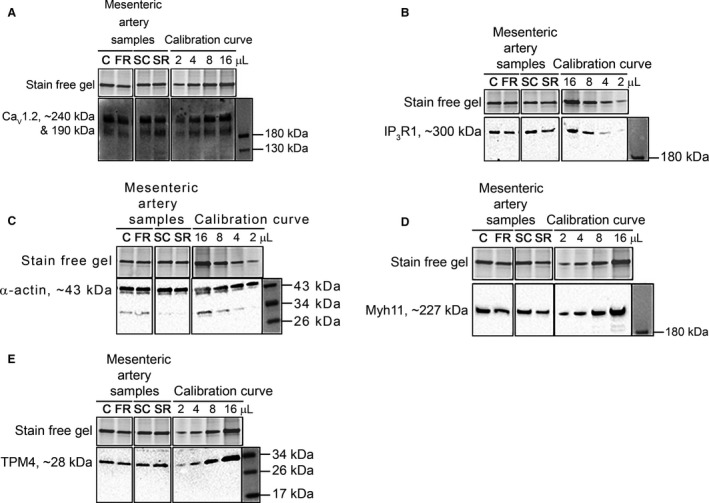
Representative western blots of the relative abundance of important contractile proteins from mesenteric artery samples of 6‐month‐old female offspring. Relative content (bottom panel) of (A) Ca_V_1.2, (B) IP_3_R1, (C) Myh11, (D) *α*‐actin and (E) TPM4 on western blot with the total protein indicated by the stain free gel (top panel) from control diet (C), food restricted (FR), surgical control (SC) and surgical restricted (SR) female offspring. Calibration curves with amount loaded (in *μ*L) specified at top in all panels. All lanes shown are from same gel. Mean ± SD values and statistical analysis displayed in Table 4.

Representative western blots investigating important contractile proteins in individual mesenteric artery samples along with respective calibration curve (e.g., 2, 4, 8 16 *μ*L), which consisted of pooled mesenteric artery samples are displayed in Figure 2 (male data) and Figure 3 (female data). Densitometric analysis of blots with mean ± SD values are shown in Table 3 (male data) and Table 4 (female data).

**Table 3:** Relative abundance of important contractile proteins from mesenteric artery samples of 6‐month‐old male offspring

	C (9)	FR (8)	SC (8)	SR (8)
Ca_V_1.2				
Total density	1.00 ± 0.10	0.48 ± 0.08***	1.00 ± 0.07	0.63 ± 0.12**
~240 kDa band	1.00 ± 0.11	0.29 ± 0.07***	1.00 ± 0.08	0.54 ± 0.12**
~190 kDa band	1.00 ± 0.49	1.08 ± 0.29	1.00 ± 0.12	1.42 ± 0.16*
IP_3_R1	1.00 ± 0.07	0.45 ± 0.14***	1.00 ± 0.08	0.54 ± 0.14**
MLCK	1.00 ± 0.12	0.48 ± 0.12**	1.00 ± 0.14	0.46 ± 0.11**
Mypt1	1.00 ± 0.11	0.56 ± 0.10**	1.00 ± 0.11	0.68 ± 0.10**
*α*‐actin	1.00 ± 0.49	1.16 ± 0.43	1.00 ± 0.16	1.60 ± 0.24*
Myh11	1.00 ± 0.30	0.84 ± 0.41	1.00 ± 0.09	0.75 ± 0.11*
TPM4	1.00 ± 0.39	0.89 ± 0.54	1.00 ± 0.23	1.14 ± 0.43

Data expressed as mean ± SD with number of individuals (*N*) shown in brackets. Significant difference (*P ≤ 0.05; **P < 0.01; ***P < 0.001; one‐tailed unpaired *t*‐test) between relevant control and restricted (C vs. FR; SC vs. SR) experimental groups.

**Table 4:** Relative abundance of important contractile proteins from mesenteric artery samples of 6‐month‐old female offspring

	C (8)	FR (11)	SC (12)	SR (10)
Ca_V_1.2				
Total density	1.00 ± 0.10	1.37 ± 0.18*	1.00 ± 0.34	0.95 ± 0.42
~240 kDa band	1.00 ± 0.37	1.43 ± 0.84	1.00 ± 0.36	0.89 ± 0.46
~190 kDa band	1.00 ± 0.13	1.48 ± 0.19*	1.00 ± 0.43	1.31 ± 0.77
IP_3_R1	1.00 ± 0.45	0.74 ± 0.46	1.00 ± 0.43	0.92 ± 0.44
*α*‐actin	1.00 ± 0.44	1.11 ± 0.52	1.00 ± 0.29	1.05 ± 0.72
Myh11	1.00 ± 0.75	0.71 ± 0.40	1.00 ± 0.39	0.98 ± 0.45
TPM4	1.00 ± 0.34	0.86 ± 0.37	1.00 ± 0.43	1.11 ± 0.51

Data expressed as mean ± SD with number of individuals (*N*) shown in brackets. Significant difference (*P ≤ 0.05; one‐tailed unpaired *t*‐test) between relevant control and restricted (C vs. FR; SC vs. SR) experimental groups.

The relative amount of the total L‐type voltage‐gated Ca^2+^ channel (Ca_V_1.2; both bands detected) was decreased in FR males compared to C (*P *<* *0.001). Analysis of individual bands revealed the FR males to have less of the large subtype (240 kDa top band) relative to the C rats (*P *<* *0.001), whereas, the abundance of the small subtype (190 kDa bottom band) was not different (*P *>* *0.05). Relative to C males, FR males had decreased amounts of IP_3_R1 (*P *<* *0.001), MLCK (*P *<* *0.01), and Mypt1 (*P *<* *0.01), whereas Myh11, *α*‐actin and TPM4 content were not different between the FR and C experimental groups.

Comparing SR and SC males, the relative total abundance of Ca_V_1.2 and specifically the large subunit (240 kDa band) was significantly (*P *< 0.01) decreased, whereas, the relative content of the small subtype (190 kDa bottom band) was significantly increased (*P *≤ 0.05). There were decreases in the relative abundances of IP_3_R1 (*P *< 0.01), MLCK (*P *< 0.01), Mypt1 (*P *< 0.01) and Myh11 (*P *≤ 0.05) was significantly decreased, whereas, *α*‐actin content was increased in SR males compared to SC (*P* ≤ 0.05).

Comparing FR females to controls, the relative total amount of Ca_V_1.2 and specifically the smaller subtype (190 kDa) was increased (*P *≤ 0.05), whereas, the relative abundance of the large subtype (240 kDa top band) of Ca_V_1.2 was unchanged. Likewise, the relative content of IP_3_R1, TPM4, Myh11 and *α*‐actin were not different (all *P* > 0.05). Furthermore, comparing SC and SR females, the relative abundance of all contractile proteins investigated were unchanged (all *P *> 0.05).

## Discussion

We have investigated the effects of two different growth restriction models on the resistance mesenteric artery of 6‐month‐old male and female WKY rats. A reduction in maternal nutrient supply from gestational day 15 to term (FR group) resulted in reduced dam growth leading to low birth weight offspring (below 10th percentile), which is consistent with previous studies (Williams et al. 2005a,b). The effects of reduced in utero blood perfusion from gestational day 18 to term (SR group), which restricts both oxygen (hypoxic) and nutrient supply, on offspring birth weight was consistent with other reports (Wlodek et al. 2008; Tare et al. 2012).

However, 6‐month‐old growth restricted male offspring had a significant reduction in maximum force when stimulated with extracellular agonists (PE and a K^+^‐induced depolarization), whereas females were unaffected. Novel findings from this study demonstrate that changes in responsiveness to extracellular agonists in growth restricted males typically reported in the literature are due to a significant reduction in maximum Ca^2+^‐activated force, with an associated reduced abundance of important contractile proteins and receptors/channels, which was not observed in growth restricted females.

### Six‐month‐old male mesenteric artery changes

Several studies have observed an altered vascular responsiveness leading to vascular dysfunction in adult offspring exposed to maternal perturbations during fetal development (Williams et al. 2005a; Anderson et al. 2006; Tare et al. 2012 to name a few). However, the underlying physiological and biochemical changes responsible remain unknown. Our results show that 6‐month‐old male growth restricted offspring (FR and SR) have a significant reduction in maximum Ca^2+^‐activated force (Table 2), which can help explain the decreased response to a K^+^‐induced depolarization and PE‐stimulation (Table 1). Activating the voltage‐ or receptor‐operated contractile pathways elevates [Ca^2+^]_i_ which subsequently increases the activity of MLCK that phosphorylates MLC leading to vasoconstriction (Barron et al. 1979; Kamm and Stull 1985). Our results show that both FR and SR 6‐month‐old males downregulated the expression of MLCK (Fig. 2C) which may contribute to the decrease in maximum Ca^2+^‐activated force (Basu et al. 2013). Gao et al. (2013) found that a 50% reduction in MLCK abundance in aortic smooth muscle resulted in a 40% inhibition in contractile response. It has also been reported that the mesenteric artery from smooth muscle specific MLCK knockout mice have reduced responsiveness to a K^+^‐induced depolarization and PE‐stimulation, and that these mice have significantly lower resting blood pressures (He et al. 2011).

Several research groups have hypothesized that the change in responsiveness to PE‐stimulation or other vasoconstrictors in growth restricted animals may be due to a shift in the abundance of key receptors (Williams et al. 2005a; Anderson et al. 2006). PE binds to the *α*_1A_‐adrenergic receptor, which produces the second messenger IP_3_, that activates its receptor (IP_3_R1) located on the internal Ca^2+^ store (sarcoplasmic reticulum) (Seasholtz et al. 1999; Sah et al. 2000). Our results show that both male 6‐month‐old growth restricted groups (FR and SR) had a significant reduction in abundance of IP_3_R1 (Fig. 2B); albeit only SR males had a significant decrease in maximum PE‐induced force response (Table 1). Tare et al. (2012) using the same SR model discovered similar results with male offspring having a significant negative shift in sensitivity to PE. IP_3_R abundance is found to be altered in several diseased states, including hypertension (Adebiyi et al. 2012; Abou‐Saleh et al. 2013) and atherosclerosis (Ewart et al. 2010). Generally, IP_3_R expression is upregulated in hypertensive models, such as genetic hypertension (Adebiyi et al. 2012) and within the spontaneously hypertensive rat model (Guillemette and Bernier 1993; Wu and de Champlain 1996), which subsequently increases PE‐induced force responses. However, during atherosclerosis which can increase oxidative stress, a phenotypic switch to a nonexcitable proproliferatory phenotype can occur; consequently, this leads to a reduction in abundance of IP_3_R1, and reduced IP_3_R‐dependent Ca^2+^ release with a dampened force response (Massaeli et al. 1999; Ewart et al. 2010). Moreover, IP_3_R1 knockout mice exhibit a blunted force response when stimulated with PE in the mesenteric artery (Santulli et al. 2017). The similar reduction in IP_3_R1 observed in this study could suggest that these rats have undergone a phenotypic switch resulting in a phenotype that is similar to an atherosclerotic state.

K^+^‐induced depolarizations activate the L‐type voltage‐operated Ca^2+^ channel (Ca_V_1.2) subsequently allowing entry of extracellular Ca^2+^ (Bolton 1979; Bülbring and Tomita 1987). Ca_V_1.2 regulates vascular resistance and thus, has a major role in regulating blood pressure (Gollasch and Nelson 1997; Sonkusare et al. 2006). The abundance of total Ca_V_1.2 was significantly reduced in both male growth restricted groups (Fig. 2A), which may help explain the decreased response to the K^+^‐induced depolarization (Table 1). Downregulation of Ca_V_1.2 through siRNA transfection (Kudryavtseva et al. 2014) or gene inactivation (Moosmang et al. 2003) is associated with a decrease in maximum response to K^+^‐induced depolarization. Ca_V_1.2 can exist as a full‐length channel (240 kDa) or be proteolytically cleaved at the C‐terminus producing a short‐truncated form (190 kDa) (De Jongh et al. 1991; Gomez‐Ospina et al. 2006; Schroder et al. 2009). The C‐terminus fragment is found to regulate the activity and expression of Ca_V_1.2 (Hulme et al. 2006; Bannister et al. 2013); therefore, the Ca_V_1.2 channel autoregulates its own expression. The C‐terminus fragment can translocate to the nucleus which increases promoter binding, reducing Ca_V_1.2 mRNA transcription and Ca_V_1.2 protein content (Schroder et al. 2009; Bannister et al. 2013). Our results show that total Ca_V_1.2 and the full‐length isoform is downregulated in both male growth restriction groups, whereas the abundance of the short‐truncated form is either unchanged (FR group) or increased (SR group) (see Table 3). These results suggest that Ca_V_1.2 is proteolytically cleaved forming the C‐terminus fragment which then inhibits the expression of Ca_V_1.2 (Schroder et al. 2009; Bannister et al. 2013) in these growth restricted male offspring. Furthermore, the C‐terminus fragment regulates the activity of Ca_V_1.2 as it reassociates at the plasma membrane and decreases the voltage sensitivity and current density of Ca_V_1.2 (Hulme et al. 2006; Bannister et al. 2013). Ca_V_1.2 can also influence gene transcription by stimulating Ca^2+^‐dependent transcriptional factors (Gollasch et al. 1998; Wamhoff et al. 2006; Kudryavtseva et al. 2014). Therefore, this excitation‐transcription coupling can affect the mesenteric arteries phenotypic expression, leading to both functional and structural changes.

### Phenotypic switching following IUGR

Studies have demonstrated that a loss of endothelial factors, coupled with a gain of proproliferatory factors due to oxidative stress, can cause a phenotypic switch leading to the development of vascular diseases, such as hypertension and atherosclerosis (Owens et al. 2004). Oxidative stress (Cambonie et al. 2007), hypertension (Ozaki et al. 2001; Alexander 2003; Brawley et al. 2003; Anderson et al. 2006; Tare et al. 2012) and endothelial dysfunction (Goodfellow et al. 1998; Leeson et al. 2001) are commonly reported to occur in arteries following IUGR, and therefore, presumably may affect these arteries phenotypic marker expression. A phenotypic switch can alter the VSMCs ability to proliferate and contract normally (Owens et al. 2004; Gomez and Owens 2012). Although phenotypic switching has been investigated in certain diseased states, this process has not been examined in VSMCs from offspring following IUGR. Specific protein marker expression (*α*‐actin, Myh11, TPM4) was examined between experimental groups, which are often used to characterize a phenotypic switch. The abundance of these specific protein markers was unchanged in both male and female FR compared to control diet groups, as well as between female SR and SC experimental groups. However, the abundance of Myh11 was significantly decreased in 6‐month‐old SR male offspring (Fig. 2F), while the abundance of *α*‐actin was increased compared to SC males (Fig. 2E). These findings would suggest that a switch from a normal contractile phenotype to a proproliferatory/migratory phenotype did not occur due to growth restriction in utero. However, it is imperative to take into consideration the abundance of Ca^2+^‐signaling proteins which regulate transcription factors, such as Ca_V_1.2 and IP_3_R1, which are often reportedly downregulated when the VSMC undergo a phenotypic switch (House et al. 2008) and can affect gene expression patterns. As stated previously, the abundance of Ca_V_1.2 and IP_3_R1 were significantly reduced in both male restricted groups (FR and SR; see Table 3), this may suggest the mesenteric artery has undergone a phenotypic switch to a more noncontractile state.

### Six‐month‐old female mesenteric artery changes

This study discovered no differences in responsiveness to either a K^+^‐induced depolarization or PE‐stimulation (Table 1) in both female FR and SR experimental groups when compared to their respective control group (C and SC). Ca^2+^‐activated forces were likewise not different (Table 2), along with the abundance of most proteins investigated (Table 4); besides an increased abundance of Ca_V_1.2 in the FR females (Fig. 3A). Results from this study support previous findings of a gender‐specific effect associated with IUGR on vascular functionality (Ozaki et al. 2001; Mazzuca et al. 2010; Bourque et al. 2013). Females seem to be protected from the negative consequences of IUGR due to the protective mechanisms of the female placenta, which has a greater resistance to excessive glucocorticoid exposure during fetal development (Murphy et al. 2002; Clifton and Murphy 2004). However, some studies have shown a change in vascular responsiveness following IUGR in female offspring (Hemmings et al. 2005; Anderson et al. 2006; Bubb et al. 2007; Sathishkumar et al. 2015). Sathishkumar et al. (2015) discovered an enhanced response to Angiotensin II (potent vasoconstrictor) in the mesenteric artery of 6‐month‐old females following a maternal protein restriction diet, however, these same arteries had no change in sensitivity to PE. Others have also shown an age‐specific difference (Hemmings et al. 2005; Anderson et al. 2006). A hypoxia‐induced growth restriction model significantly affected myogenic tone at 4‐months of age in females, whereas at 7‐months, no functional differences were found (Hemmings et al. 2005). Results may vary between the different studies due to the differences in specific age group investigated, or the severity and timing of the diverse growth restriction models employed. Innate sex‐specific differences in the regulation of several biological systems, such as the renin–angiotensin system (Woods et al. 2001, 2005), regulation of oxidative stress (Katkhuda et al. 2012; Ojeda et al. 2012) and blood pressure (Ojeda et al. 2007a,b), may contribute to the disparity in results between males and females in this study.

## Conclusion

In conclusion, this is the first study to discover that the decrease in vascular responsiveness in IUGR males is caused by a reduced maximum Ca^2+^‐activated force response and likely contributed by the decreased abundance of important contractile proteins activated in the excitation‐contraction coupling pathways. Furthermore, the decrease in Ca^2+^‐signaling proteins and receptors/channels (Ca_V_1.2 and IP_3_R1) may contribute to the reduced contractility by possibly prompting a phenotypic switch to a more noncontractile phenotypic state. From this study, it was clear there was a sex‐specific disparity in the vascular changes associated with IUGR with restricted females showing no changes in contractile responsiveness to extracellular agonists, or direct Ca^2+^‐activated force, and generally no difference in the abundance of important contractile proteins.

## References

[phy213954-bib-0001] Abou‐Saleh, H. , A. R. Pathan , A. Daalis , S. Hubrack , H. Abou‐Jassoum , H. Al‐Naeimi , et al. 2013 Inositol 1,4,5‐trisphosphate (IP_3_) receptor up‐regulation in hypertension is associated with sensitization of Ca^2+^ release and vascular smooth muscle contractility. J. Biol. Chem. 288:32941–32951. 24097979 10.1074/jbc.M113.496802 PMC3829145

[phy213954-bib-0002] Adebiyi, A. , C. M. Thomas‐Gatewood , M. D. Leo , M. W. Kidd , Z. P. Neeb , and J. H. Jaggar . 2012 An elevation in physical coupling of type 1 inositol 1,4,5‐trisphosphate (IP_3_) receptors to transient receptor potential 3 (TRPC3) channels constricts mesenteric arteries in genetic hypertension. Hypertension 60:1213–1219. 23045459 10.1161/HYPERTENSIONAHA.112.198820 PMC3632264

[phy213954-bib-0003] Akata, T. , and W. A. III Boyle . 1997 Is guanosine‐5’’‐triphosphate Involved in calcium‐activation of contractile proteins in vascular smooth muscle? Jpn. J. Pharmacol. 75:1–12. 9334880 10.1254/jjp.75.1

[phy213954-bib-0004] Alexander, B. T. 2003 Placental insufficiency leads to development of hypertension in growth‐restricted offspring. Hypertension 41:457–462. 12623943 10.1161/01.HYP.0000053448.95913.3D

[phy213954-bib-0005] Anderson, C. M. , F. Lopez , A. Zimmer , and J. N. Benoit . 2006 Placental insufficiency leads to developmental hypertension and mesenteric artery dysfunction in two generations of Sprague‐Dawley rat offspring. Biol. Reprod. 74:538–544. 16306423 10.1095/biolreprod.105.045807

[phy213954-bib-0006] Bannister, J. P. , M. D. Leo , D. Narayanan , W. Jangsangthong , A. Nair , K. W. Evanson , et al. 2013 The voltage‐dependent L‐type Ca^2+^ (Ca_V_1.2) channel C‐terminus fragment is a bi‐modal vasodilator. J. Physiol. 591:2987–2998. 23568894 10.1113/jphysiol.2013.251926 PMC3832115

[phy213954-bib-0007] Barker, D. 1994 Outcome of low birthweight. Horm. Res. Paediatr. 42:223–230. 10.1159/000184197 7868077

[phy213954-bib-0008] Barron, J. T. , M. Barany , and K. Barany . 1979 Phosphorylation of the 20,000‐dalton light chain of myosin of intact arterial smooth muscle in rest and in contraction. J. Biol. Chem. 254:4954–4956. 447626

[phy213954-bib-0009] Basu, S. , D. K. Srinivasan , K. Yang , H. Raina , S. Banerjee , R. Zhang , et al. 2013 Notch transcriptional control of vascular smooth muscle regulatory gene expression and function. J. Biol. Chem. 288:11191–11202. 23482558 10.1074/jbc.M112.442996 PMC3630855

[phy213954-bib-0010] Bergmann, R. , K. Bergmann , and J. Dudenhausen . 2008 Undernutrition and growth restriction in pregnancy Pp. 103–121 in Barker D. J. P., Bergmann R. L. and Ogra P. L., eds. The window of opportunity: pre‐pregnancy to 24 months of age. Karger Publishers, Basel.

[phy213954-bib-0011] Bolton, T. 1979 Mechanisms of action of transmitters and other substances on smooth muscle. Physiol. Rev. 59:606–718. 37533 10.1152/physrev.1979.59.3.606

[phy213954-bib-0012] Bourque, S. L. , F. S. Gragasin , A. L. Quon , Y. Mansour , J. S. Morton , and S. T. Davidge . 2013 Prenatal hypoxia causes long‐term alterations in vascular endothelin‐1 function in aged male, but not female, offspring. Hypertension 62:753–758. 23940196 10.1161/HYPERTENSIONAHA.113.01516

[phy213954-bib-0013] Brawley, L. , S. Itoh , C. Torrens , A. Barker , C. Bertram , L. Poston , et al. 2003 Dietary protein restriction in pregnancy induces hypertension and vascular defects in rat male offspring. Pediatr. Res. 54:83–90. 12646717 10.1203/01.PDR.0000065731.00639.02

[phy213954-bib-0014] Bubb, K. J. , M. L. Cock , M. J. Black , M. Dodic , W. M. Boon , H. C. Parkington , et al. 2007 Intrauterine growth restriction delays cardiomyocyte maturation and alters coronary artery function in the fetal sheep. J. Physiol. 578:871–881. 17124269 10.1113/jphysiol.2006.121160 PMC2151351

[phy213954-bib-0015] Bülbring, E. , and T. Tomita . 1987 Catecholamine action on smooth muscle. Pharmacol. Rev. 39:49–96. 3033708

[phy213954-bib-0016] Cambonie, G. , B. Comte , C. Yzydorczyk , T. Ntimbane , N. Germain , N. L. O. Lê , et al. 2007 Antenatal antioxidant prevents adult hypertension, vascular dysfunction, and microvascular rarefaction associated with in utero exposure to a low‐protein diet. Am. J. Physiol. Regul. Integr. Comp. Physiol. 292:R1236–R1245. 17138729 10.1152/ajpregu.00227.2006

[phy213954-bib-0017] Christie, M . 2018 The effect of intrauterine growth restriction on Ca^2+^‐activated force and contractile protein expression in the mesenteric artery of young, adult and aged Wistar‐Kyoto rats. PhD Thesis, La Trobe University. 10.1007/s13105-020-00724-6 31927696

[phy213954-bib-0018] Clifton, V. , and V. Murphy . 2004 Maternal asthma as a model for examining fetal sex‐ specific effects on maternal physiology and placental mechanisms that regulate human fetal growth. Placenta 25:S45–S52. 15033307 10.1016/j.placenta.2004.01.004

[phy213954-bib-0019] De Jongh, K. S. , C. Warner , A. A. Colvin , and W. A. Catterall . 1991 Characterization of the two size forms of the alpha 1 subunit of skeletal muscle L‐type calcium channels. Proc. Natl. Acad. Sci. 88:10778–10782. 1720551 10.1073/pnas.88.23.10778 PMC53014

[phy213954-bib-0020] Edwards, J. N. , O. Friedrich , T. R. Cully , F. von Wegner , R. M. Murphy , and B. S. Launikonis . 2010 Upregulation of store‐operated Ca^2+^ entry in dystrophic mdx mouse muscle. Am. J. Physiol. Cell Physiol. 299:C42–C50. 20427714 10.1152/ajpcell.00524.2009

[phy213954-bib-0021] Ewart, M.‐A. , J. G. McCarron , S. Kennedy , and S. Currie . 2010 SERCA and IP3R expression and function in vascular smooth muscle is altered throughout atherosclerotic progression. Biophys. J. 98:677a.

[phy213954-bib-0022] Gao, N. , J. Huang , W. He , M. Zhu , K. E. Kamm , and J. T. Stull . 2013 Signaling through myosin light chain kinase in smooth muscles. J. Biol. Chem. 288:7596–7605. 23362260 10.1074/jbc.M112.427112 PMC3597801

[phy213954-bib-0023] Gluckman, P. D. , and M. A. Hanson . 2004 Developmental origins of disease paradigm: a mechanistic and evolutionary perspective. Pediatr. Res. 56:311–317. 15240866 10.1203/01.PDR.0000135998.08025.FB

[phy213954-bib-0024] Gollasch, M. , and M. T. Nelson . 1997 Voltage‐dependent Ca^2+^ channels in arterial smooth muscle cells. Kidney Blood Press. Res. 20:355–371. 9453447 10.1159/000174250

[phy213954-bib-0025] Gollasch, M. , H. Haase , C. Ried , C. Lindschau , I. Morano , F. C. Luft , et al. 1998 L‐type calcium channel expression depends on the differentiated state of vascular smooth muscle cells. FASEB J. 12:593–601. 9576486 10.1096/fasebj.12.7.593

[phy213954-bib-0026] Gomez, D. , and G. K. Owens . 2012 Smooth muscle cell phenotypic switching in atherosclerosis. Cardiovasc. Res. 95:156–164. 22406749 10.1093/cvr/cvs115 PMC3388816

[phy213954-bib-0027] Gomez‐Ospina, N. , F. Tsuruta , O. Barreto‐Chang , L. Hu , and R. Dolmetsch . 2006 The C terminus of the L‐type voltage‐gated calcium channel Ca_V_1.2 encodes a transcription factor. Cell 127:591–606. 17081980 10.1016/j.cell.2006.10.017 PMC1750862

[phy213954-bib-0028] Goodfellow, J. , M. F. Bellamy , S. T. Gorman , M. Brownlee , M. W. Ramsey , M. J. Lewis , et al. 1998 Endothelial function is impaired in fit young adults of low birth weight. Cardiovasc. Res. 40:600–606. 10070502 10.1016/s0008-6363(98)00197-7

[phy213954-bib-0029] Guillemette, G. , and S. Bernier . 1993 Increased inositol 1, 4, 5‐trisphosphate binding capacity in vascular smooth muscle of spontaneously hypertensive rats. Am. J. Hypertens. 6:217–225. 8466709

[phy213954-bib-0030] Harvey, T. J. , R. M. Murphy , J. L. Morrison , and G. S. Posterino . 2015 Maternal nutrient restriction alters Ca2 + handling properties and contractile function of isolated left ventricle bundles in male but not female juvenile rats. PLoS ONE 10:e0138388. 26406887 10.1371/journal.pone.0138388 PMC4583465

[phy213954-bib-0031] He, W. Q. , Y. N. Qiao , C. H. Zhang , Y. J. Peng , C. Chen , P. Wang , et al. 2011 Role of myosin light chain kinase in regulation of basal blood pressure and maintenance of salt‐induced hypertension. Am. J. Physiol. Heart Circ. Physiol. 301:H584–H591. 21572007 10.1152/ajpheart.01212.2010 PMC3154661

[phy213954-bib-0032] Hemmings, D. G. , S. J. Williams , and S. T. Davidge . 2005 Increased myogenic tone in 7‐month‐old adult male but not female offspring from rat dams exposed to hypoxia during pregnancy. Am. J. Physiol. Heart Circ. Physiol. 289:H674–H682. 15833805 10.1152/ajpheart.00191.2005

[phy213954-bib-0033] Henriksen, T. , and T. Clausen . 2002 The fetal origins hypothesis: placental insufficiency and inheritance versus maternal malnutrition in well‐nourished populations. Acta Obstet. Gynecol. Scand. 81:112–114. 11942899 10.1034/j.1600-0412.2002.810204.x

[phy213954-bib-0034] House, S. J. , M. Potier , J. Bisaillon , H. A. Singer , and M. Trebak . 2008 The non‐excitable smooth muscle: calcium signaling and phenotypic switching during vascular disease. Pflugers Arch. 456:769–785. 18365243 10.1007/s00424-008-0491-8 PMC2531252

[phy213954-bib-0035] Hulme, J. T. , V. Yarov‐Yarovoy , T. W. Lin , T. Scheuer , and W. A. Catterall . 2006 Autoinhibitory control of the CaV1.2 channel by its proteolytically processed distal C‐terminal domain. J. Physiol. 576:87–102. 16809371 10.1113/jphysiol.2006.111799 PMC1995633

[phy213954-bib-0036] Hungerford, J. , and C. Little . 1999 Developmental biology of the vascular smooth muscle cell: building a multilayered vessel wall. J. Vasc. Res. 36:2–27. 10050070 10.1159/000025622

[phy213954-bib-0037] Jain, R. K. 2003 Molecular regulation of vessel maturation. Nat. Med. 9:685–693. 12778167 10.1038/nm0603-685

[phy213954-bib-0038] Kamm, K. E. , and J. T. Stull . 1985 The function of myosin and myosin light chain kinase phosphorylation in smooth muscle. Annu. Rev. Pharmacol. Toxicol. 25:593–620. 2988424 10.1146/annurev.pa.25.040185.003113

[phy213954-bib-0039] Katkhuda, R. , E. S. Peterson , R. D. Roghair , A. W. Norris , T. D. Scholz , and J. L. Segar . 2012 Sex‐specific programming of hypertension in offspring of late gestation diabetic rats. Pediatr. Res. 72:352. 22805998 10.1038/pr.2012.93 PMC3607358

[phy213954-bib-0040] Khong, T. , F. Wolf , W. Robertson , and I. Brosens . 1986 Inadequate maternal vascular response to placentation in pregnancies complicated by pre‐eclampsia and by small‐for‐gestational age infants. BJOG 93:1049–1059. 10.1111/j.1471-0528.1986.tb07830.x 3790464

[phy213954-bib-0041] Khorram, O. , M. Momeni , M. Desai , and M. G. Ross . 2007 Nutrient restriction in utero induces remodeling of the vascular extracellular matrix in rat offspring. Reprod. Sci. 14:73–80. 17636219 10.1177/1933719106298215

[phy213954-bib-0042] Kitazawa, T. , and A. P. Somlyo . 1990 Desensitization and muscarinic re‐sensitization of force and myosin light chain phosphorylation to cytoplasmic Ca^2+^ in smooth muscle. Biochem. Biophys. Res. Commun. 172:1291–1297. 2244912 10.1016/0006-291x(90)91589-k

[phy213954-bib-0043] Kitazawa, T. , M. Masuo , and A. P. Somlyo . 1991 G protein‐mediated inhibition of myosin light‐chain phosphatase in vascular smooth muscle. Proc. Indian Natl. Sci. 88:9307–9310. 10.1073/pnas.88.20.9307 PMC52703 1656467

[phy213954-bib-0044] Kudryavtseva, O. , K. M. Herum , V. S. Dam , M. S. Straarup , D. Kamaev , D. M. Briggs Boedtkjer , et al. 2014 Downregulation of L‐type Ca^2+^ channel in rat mesenteric arteries leads to loss of smooth muscle contractile phenotype and inward hypertrophic remodeling. Am. J. Physiol. Heart Circ. Physiol. 306:H1287–H1301. 24561864 10.1152/ajpheart.00503.2013

[phy213954-bib-0045] Lamb, G. , and D. Stephenson . 1990 Control of calcium release and the effect of ryanodine in skinned muscle fibres of the toad. J. Physiol. 423:519–542. 2167367 10.1113/jphysiol.1990.sp018037 PMC1189772

[phy213954-bib-0046] Lamb, G. , and D. Stephenson . 1994 Effects of intracellular pH and [Mg^2+^] on excitation‐contraction coupling in skeletal muscle fibres of the rat. J. Physiol. 478:331–339. 7965848 10.1113/jphysiol.1994.sp020253 PMC1155689

[phy213954-bib-0047] Leeson, C. , M. Kattenhorn , R. Morley , A. Lucas , and J. Deanfield . 2001 Impact of low birth weight and cardiovascular risk factors on endothelial function in early adult life. Circulation 103:1264–1268. 11238271 10.1161/01.cir.103.9.1264

[phy213954-bib-0048] MacInnis, M. J. , E. Zacharewicz , B. J. Martin , M. E. Haikalis , L. E. Skelly , M. A. Tarnopolsky , et al. 2017 Superior mitochondrial adaptations in human skeletal muscle after interval compared to continuous single‐leg cycling matched for total work. J. Physiol. 595:2955–2968. 27396440 10.1113/JP272570 PMC5407978

[phy213954-bib-0049] Massaeli, H. , J. A. Austria , and G. N. Pierce . 1999 Chronic exposure of smooth muscle cells to minimally oxidized LDL results in depressed inositol 1, 4, 5‐trisphosphate receptor density and Ca^2+^ transients. Circ. Res. 85:515–523. 10488054 10.1161/01.res.85.6.515

[phy213954-bib-0050] Mazzuca, M. Q. , M. E. Wlodek , N. M. Dragomir , H. C. Parkington , and M. Tare . 2010 Uteroplacental insufficiency programs regional vascular dysfunction and alters arterial stiffness in female offspring. J. Physiol. 588:1997–2010. 20403978 10.1113/jphysiol.2010.187849 PMC2901985

[phy213954-bib-0051] McIntyre, C. , B. Williams , R. Lindsay , J. McKnight , and P. Hadoke . 1998 Preservation of vascular function in rat mesenteric resistance arteries following cold storage, studied by small vessel myography. Br. J. Pharmacol. 123:1555–1560. 9605561 10.1038/sj.bjp.0701768 PMC1565325

[phy213954-bib-0052] McMillen, I. C. , and J. S. Robinson . 2005 Developmental origins of the metabolic syndrome: prediction, plasticity, and programming. Physiol. Rev. 85:571–633. 15788706 10.1152/physrev.00053.2003

[phy213954-bib-0053] Mollica, J. P. , J. S. Oakhill , G. D. Lamb , and R. M. Murphy . 2009 Are genuine changes in protein expression being overlooked? Reassessing Western blotting. Anal. Biochem. 386:270–275. 19161968 10.1016/j.ab.2008.12.029

[phy213954-bib-0054] Moosmang, S. , V. Schulla , A. Welling , R. Feil , S. Feil , J. W. Wegener , et al. 2003 Dominant role of smooth muscle L‐type calcium channel Ca v 1.2 for blood pressure regulation. EMBO J. 22:6027–6034. 14609949 10.1093/emboj/cdg583 PMC275441

[phy213954-bib-0055] Mulvany, M. J. , and W. Halpern . 1977 Contractile properties of small arterial resistance vessels in spontaneously hypertensive and normotensive rats. Circ. Res. 41:19–26. 862138 10.1161/01.res.41.1.19

[phy213954-bib-0056] Murphy, R. M. , and G. D. Lamb . 2013 Important considerations for protein analyses using antibody based techniques: down‐sizing Western blotting up‐sizes outcomes. J. Physiol. 591:5823–5831. 24127618 10.1113/jphysiol.2013.263251 PMC3872754

[phy213954-bib-0057] Murphy, V. E. , T. Zakar , R. Smith , W. B. Giles , P. G. Gibson , and V. L. Clifton . 2002 Reduced 11*β*‐hydroxysteroid dehydrogenase type 2 activity is associated with decreased birth weight centile in pregnancies complicated by asthma. J. Clin. Endocrinol. Metab. 87:1660–1668. 11932298 10.1210/jcem.87.4.8377

[phy213954-bib-0058] Nusken, K. D. , J. Dotsch , M. Rauh , W. Rascher , and H. Schneider . 2008 Uteroplacental insufficiency after bilateral uterine artery ligation in the rat: impact on postnatal glucose and lipid metabolism and evidence for metabolic programming of the offspring by sham operation. Endocrinology 149:1056–1063. 18063678 10.1210/en.2007-0891

[phy213954-bib-0059] O'Dowd, R. , J. C. Kent , J. M. Moseley , and M. E. Wlodek . 2008 Effects of uteroplacental insufficiency and reducing litter size on maternal mammary function and postnatal offspring growth. Am. J. Physiol. Regul. Integr. Comp. Physiol. 294:R539–R548. 18077510 10.1152/ajpregu.00628.2007

[phy213954-bib-0060] Ojeda, N. B. , D. Grigore , L. L. Yanes , R. Iliescu , E. B. Robertson , H. Zhang , et al. 2007a Testosterone contributes to marked elevations in mean arterial pressure in adult male intrauterine growth restricted offspring. Am. J. Physiol. Regul. Integr. Comp. Physiol. 292:R758–R763. 16917022 10.1152/ajpregu.00311.2006

[phy213954-bib-0061] Ojeda, N. B. , D. Grigore , E. B. Robertson , and B. T. Alexander . 2007b Estrogen protects against increased blood pressure in postpubertal female growth restricted offspring. Hypertension 50:679–685. 17724277 10.1161/HYPERTENSIONAHA.107.091785 PMC2850594

[phy213954-bib-0062] Ojeda, N. B. , B. S. Hennington , D. T. Williamson , M. L. Hill , N. E. Betson , J. C. Sartori‐Valinotti , et al. 2012 Oxidative stress contributes to sex differences in blood pressure in adult growth‐restricted offspring novelty and significance. Hypertension 60:114–122. 22585945 10.1161/HYPERTENSIONAHA.112.192955 PMC3655434

[phy213954-bib-0063] Owens, G. K. 1995 Regulation of differentiation of vascular smooth muscle cells. Physiol. Rev. 75:487–517. 7624392 10.1152/physrev.1995.75.3.487

[phy213954-bib-0064] Owens, G. K. , M. S. Kumar , and B. R. Wamhoff . 2004 Molecular regulation of vascular smooth muscle cell differentiation in development and disease. Physiol. Rev. 84:767–801. 15269336 10.1152/physrev.00041.2003

[phy213954-bib-0065] Ozaki, T. , H. Nishina , M. Hanson , and L. Poston . 2001 Dietary restriction in pregnant rats causes gender‐related hypertension and vascular dysfunction in offspring. J. Physiol. 530:141–152. 11136866 10.1111/j.1469-7793.2001.0141m.x PMC2278385

[phy213954-bib-0066] Peleg, D. , C. M. Kennedy , and S. K. Hunter . 1998 Intrauterine growth restriction: identification and management. Am. Fam. Physician 58:457–466. 9713399

[phy213954-bib-0067] Posterino, G. S. , G. D. Lamb , and D. G. Stephenson . 2000 Twitch and tetanic force responses and longitudinal propagation of action potentials in skinned skeletal muscle fibres of the rat. J. Physiol. 527:131–137. 10944176 10.1111/j.1469-7793.2000.t01-2-00131.x PMC2270051

[phy213954-bib-0068] Sah, V. P. , T. M. Seasholtz , S. A. Sagi , and J. H. Brown . 2000 The role of Rho in G protein‐coupled receptor signal transduction. Annu. Rev. Pharmacol. Toxicol. 40:459–489. 10836144 10.1146/annurev.pharmtox.40.1.459

[phy213954-bib-0069] Salmon, M. , D. Gomez , E. Greene , L. Shankman , and G. K. Owens . 2012 Cooperative binding of KLF4, pELK‐1, and HDAC2 to a G/C repressor element in the SM22*α* promoter mediates transcriptional silencing during SMC phenotypic switching in vivo novelty and significance. Circ. Res. 111:685–696. 22811558 10.1161/CIRCRESAHA.112.269811 PMC3517884

[phy213954-bib-0070] Santulli, G. , J. Gambardella , S. Reiken , Q. Yuan , R. Nakashima , F. M. Forrester , et al. 2017 Mechanistic role of type 1 inositol 1,4,5‐trisphosphate receptor in the regulation of vascular tone in heart failure. Biophys. J. 112:482a.

[phy213954-bib-0071] Sathishkumar, K. , M. P. Balakrishnan , and C. Yallampalli . 2015 Enhanced mesenteric arterial responsiveness to angiotensin II is androgen receptor‐dependent in prenatally protein‐restricted adult female rat offspring. Biol. Reprod. 92:55. 25550341 10.1095/biolreprod.114.126482 PMC4342791

[phy213954-bib-0072] Satoh, S. , R. Kreutz , C. Wilm , D. Ganten , and G. Pfitzer . 1994 Augmented agonist‐induced Ca (2 + )‐sensitization of coronary artery contraction in genetically hypertensive rats. Evidence for altered signal transduction in the coronary smooth muscle cells. J. Clin. Investig. 94:1397–1403. 7929815 10.1172/JCI117475 PMC295265

[phy213954-bib-0073] Schroder, E. , M. Byse , and J. Satin . 2009 L‐type calcium channel C terminus autoregulates transcription. Circ. Res. 104:1373–1381. 19461046 10.1161/CIRCRESAHA.108.191387 PMC2728034

[phy213954-bib-0074] Seasholtz, T. M. , M. Majumdar , and J. H. Brown . 1999 Rho as a mediator of G protein‐coupled receptor signaling. Mol. Pharmacol. 55:949–956. 10347235 10.1124/mol.55.6.949

[phy213954-bib-0075] Somlyo, A. P. , and A. V. Somlyo . 2003 Ca^2+^ sensitivity of smooth muscle and nonmuscle myosin II: modulated by G proteins, kinases, and myosin phosphatase. Physiol. Rev. 83:1325–1358. 14506307 10.1152/physrev.00023.2003

[phy213954-bib-0076] Sonkusare, S. , P. T. Palade , J. D. Marsh , S. Telemaque , A. Pesic , and N. J. Rusch . 2006 Vascular calcium channels and high blood pressure: pathophysiology and therapeutic implications. Vascul. Pharmacol. 44:131–142. 16427812 10.1016/j.vph.2005.10.005 PMC4917380

[phy213954-bib-0077] Stephenson, D. , and D. Williams . 1981 Calcium‐activated force responses in fast‐and slow‐ twitch skinned muscle fibres of the rat at different temperatures. J. Physiol. 317:281–302. 7310735 10.1113/jphysiol.1981.sp013825 PMC1246789

[phy213954-bib-0078] Tare, M. , H. C. Parkington , K. J. Bubb , and M. E. Wlodek . 2012 Uteroplacental insufficiency and lactational environment separately influence arterial stiffness and vascular function in adult male rats. Hypertension 60:378–386. 22733472 10.1161/HYPERTENSIONAHA.112.190876

[phy213954-bib-0079] Wadley, G. D. , A. L. Siebel , G. J. Cooney , G. K. McConell , M. E. Wlodek , and J. A. Owens . 2008 Uteroplacental insufficiency and reducing litter size alters skeletal muscle mitochondrial biogenesis in a sex‐specific manner in the adult rat. Am. J. Physiol. Endocrinol. Metab. 294:E861–E869. 18319353 10.1152/ajpendo.00037.2008

[phy213954-bib-0080] Wamhoff, B. R. , D. K. Bowles , and G. K. Owens . 2006 Excitation‐transcription coupling in arterial smooth muscle. Circ. Res. 98:868–878. 16614312 10.1161/01.RES.0000216596.73005.3c

[phy213954-bib-0081] Williams, S. J. , M. E. Campbell , I. C. McMillen , and S. T. Davidge . 2005a Differential effects of maternal hypoxia or nutrient restriction on carotid and femoral vascular function in neonatal rats. Am. J. Physiol. Regul. Integr. Comp. Physiol. 288:R360–R367. 15528396 10.1152/ajpregu.00178.2004

[phy213954-bib-0082] Williams, S. J. , D. G. Hemmings , J. M. Mitchell , I. C. McMillen , and S. T. Davidge . 2005b Effects of maternal hypoxia or nutrient restriction during pregnancy on endothelial function in adult male rat offspring. J. Physiol. 565:125–135. 15774515 10.1113/jphysiol.2005.084889 PMC1464495

[phy213954-bib-0083] Williams, D. W. , D. G. Stephenson , and G. S. Posterino . 2017 The effects of Suramin on Ca2 + activated force and sarcoplasmic reticulum Ca2 + release in skinned fast‐twitch skeletal muscle fibers of the rat. Physiol. Rep. 5:e13333. 28743820 10.14814/phy2.13333 PMC5532480

[phy213954-bib-0084] Wlodek, M. , K. Westcott , A. Serruto , R. O'Dowd , L. Wassef , P. Ho , et al. 2003 Impaired mammary function and parathyroid hormone‐related protein during lactation in growth‐restricted spontaneously hypertensive rats. J. Endocrinol. 178:233–245. 12904171 10.1677/joe.0.1780233

[phy213954-bib-0085] Wlodek, M. E. , K. T. Westcott , R. O'Dowd , A. Serruto , L. Wassef , K. M. Moritz , et al. 2005 Uteroplacental restriction in the rat impairs fetal growth in association with alterations in placental growth factors including PTHrP. Am. J. Physiol. Regul. Integr. Comp. Physiol. 288:R1620–R1627. 15661964 10.1152/ajpregu.00789.2004

[phy213954-bib-0086] Wlodek, M. E. , A. Mibus , A. Tan , A. L. Siebel , J. A. Owens , and K. M. Moritz . 2007 Normal lactational environment restores nephron endowment and prevents hypertension after placental restriction in the rat. J. Am. Soc. Nephrol. 18:1688–1696. 17442788 10.1681/ASN.2007010015

[phy213954-bib-0087] Wlodek, M. E. , K. Westcott , A. L. Siebel , J. A. Owens , and K. M. Moritz . 2008 Growth restriction before or after birth reduces nephron number and increases blood pressure in male rats. Kidney Int. 74:187–195. 18432184 10.1038/ki.2008.153

[phy213954-bib-0088] Woods, L. L. , J. R. Ingelfinger , J. R. Nyengaard , and R. Rasch . 2001 Maternal protein restriction suppresses the newborn renin‐angiotensin system and programs adult hypertension in rats. Pediatr. Res. 49:460–467. 11264427 10.1203/00006450-200104000-00005

[phy213954-bib-0089] Woods, L. L. , J. R. Ingelfinger , and R. Rasch . 2005 Modest maternal protein restriction fails to program adult hypertension in female rats. Am. J. Physiol. Regul. Integr. Comp. Physiol. 289:R1131–R1136. 15961538 10.1152/ajpregu.00037.2003

[phy213954-bib-0090] Wu, L. , and J. de Champlain . 1996 Inhibition by cyclic AMP of basal and induced inositol phosphate production in cultured aortic smooth muscle cells from Wistar‐Kyoto and spontaneously hypertensive rats. J. Hypertens. 14:593–599. 8762202 10.1097/00004872-199605000-00008

[phy213954-bib-0091] Yoshida, T. , K. H. Kaestner , and G. K. Owens . 2008 Conditional deletion of Krüppel‐like factor 4 delays downregulation of smooth muscle cell differentiation markers but accelerates neointimal formation following vascular injury. Circ. Res. 102:1548–1557. 18483411 10.1161/CIRCRESAHA.108.176974 PMC2633447

